# 

**Published:** 1874-09

**Authors:** 


### Contents of September Number.

### ORIGINAL DEPARTMENT.

ART. I.—The Relations of General Medicine to Specific Modes of Medication. By Alfred Mercer, M. D., Syracuse, N. Y. .............. 41

ART. II.—Medical Society of the County of Albany. Semi-Monthly Meeting, May 13. Reported by F. C. Curtis, M. D., Secretary.... 58

ART. III.—Abstract of the Proceedings of the Buffalo Medical Association, August 4, 1874. Reported by Jos. Fowler, M. D., Secretary, Pro. Tem. ... .............................................. 66

ART. IV.—Semi-Monthly Meeting, Rochester Medical Society. Reported by J. O. Roe, M. D., Secretary.......................... 73

### EDITORIAL DEPARTMENT.

Causes of Disease in large Cities.............................. 74

OBITUARY.

Dr. J. N. Brown, Buffalo ............................ 75

Prof. Jeffries Wyman, Boston ............................ 76

Items.............................................. 76

BOOKS REVIEWED :

The Physiology of Man, Vol. V. By Austin Flint, Jr., M.D. 77

A Treatise on Diseases of the Genito-Urinary Organs. By W. H. Van Buren, M. D., and E. L. Keyes, M. D....... 79

Books and Pamphlets Received...... .. ........................ 80

THE BUFFALO

